# Determining the amphipol distribution within membrane-protein fibre samples using small-angle neutron scattering

**DOI:** 10.1107/S205979831800476X

**Published:** 2018-11-30

**Authors:** Wanatchaporn Arunmanee, Richard K. Heenan, Jeremy H. Lakey

**Affiliations:** a Department of Biochemistry and Microbiology, Faculty of Pharmaceutical Sciences, Chulalongkorn University, Bangkok 10330, Thailand; b Institute for Cell and Molecular Bioscience, Newcastle University, Framlington Place, Newcastle upon Tyne NE2 4HH, England; c ISIS Pulsed Neutron and Muon Source, STFC Rutherford Appleton Laboratory, Didcot OX11 0QX, England

**Keywords:** amphipol, membrane proteins, small-angle neutron scattering, deuteration

## Abstract

Detergent micelles can solubilize membrane proteins, but there is always a need for a pool of free detergent at the critical micellar concentration to maintain the micelle–monomer equilibrium. Amphipol polymeric surfactants (APols) have been developed to replace conventional detergents in membrane-protein studies, but the role of free amphipol is unclear. It has previously been shown that the removal of free APol causes monodisperse outer membrane protein F (OmpF) to form long filaments. However, any remaining APol could not be resolved using electron microscopy. Here, small-angle neutron scattering with isotope contrast matching was used to separately determine the distributions of membrane protein and amphipol in a mixed sample. The data showed that after existing free amphipol had been removed from monodisperse complexes, a new equilibrium was established between protein–amphipol filaments and a pool of newly liberated free amphipol. The filaments consisted of OmpF proteins surrounded by a belt of Apol, whilst free oblate spheroid micelles of Apol were also present. No indications of long-range order were observed, suggesting a lack of defined structure in the filaments.

## Abstract 2

Using the unique ability of small-angle neutron scattering to resolve a hydrogen-rich surfactant from a deuterated membrane protein, the results of removing free surfactant from the equilibrium are revealed.

## 1. Introduction   

Membrane proteins (MPs) play a vital role in cell function, and many of them, such as GPCRs and ion channels, have been exploited as drug targets. Therefore, over the years they have been the target of many structural and functional studies. Conventionally, when extracting MPs from biological membranes they must be handled in detergents in order to keep them soluble in aqueous solution. As detergents sometimes destabilize MPs, it is a formidable task to look for suitable detergents which maintain both their structure and function. To overcome this problem, several novel approaches have been developed to stabilize MPs in close-to-native environments (Hein *et al.*, 2014 ▸). J.-L. Popot and coworkers invented a new class of detergents which are based upon an amphipathic polymer called ‘amphipol’ (APol; Tribet *et al.*, 1996 ▸). APol comprises an anionic polyacrylate backbone partially and randomly derivatized with hydrophobic groups: octylamine and isopropylamine. APol makes multiple contacts with MPs, hence the affinity of MP for APol is high. In contrast to conventional detergents, APol is able to solubilize MPs in the near-absence of free APol (Tribet *et al.*, 1997 ▸; Popot *et al.*, 2003 ▸). Structural studies of MP in complex with APol have been carried out using several biophysical techniques such as electron microscopy (EM; see, for example, Cao *et al.*, 2013 ▸; Liao *et al.*, 2013 ▸; Lu *et al.*, 2014 ▸; Fitzpatrick *et al.*, 2017 ▸), small-angle neutron scattering (SANS; Gohon *et al.*, 2008 ▸) and nuclear magnetic resonance (NMR; Zoonens *et al.*, 2005 ▸; Catoire *et al.*, 2010 ▸).

Several studies have shown that APol improves the stability of both the α-helical and β-barrel types of MPs (Kleinschmidt & Popot, 2014 ▸). Heat denaturation of bacteriorhodopsins (BRs) in the absence and presence of APol has been observed. BRs were more stable at high temperature in APol than in *n*-octyl-β-thioglucoside (Dahmane *et al.*, 2013 ▸). APol has also been shown to enhance the thermostability of the GPCR leukotriene B4 receptor (BLT1) in comparison to mixed micelles (Dahmane *et al.*, 2009 ▸). The stability of β-barrel MPs was tested under high-temperature or chemical denaturing conditions. This illustrated that the major outer membrane protein from the pathogenic bacterium *Chlamydia trachomatis* does not unfold in APol until the temperature reaches 78°C (Tifrea *et al.*, 2011 ▸). OmpA, the outer membrane protein from *Escherichia coli*, was more resistant to denaturation by urea in APol compared with LDAO (Pocanschi *et al.*, 2013 ▸).

Even though APol can stabilize MPs in solution, the approach used for the preparation of MP–APol complexes can have an effect on their stability. For example, it has been reported that the removal of free APol from solutions of MP–APol complexes leads to self-association of the complexes. An initially homogenous state of MP–APol complexes became heterogeneous when depleted of free APol (Zoonens *et al.*, 2007 ▸). Likewise, self-organization of BR–APol and OmpF–APol into long filaments was observed by EM when the preparation of these complexes was performed using an approach which completely removed free APol (Gohon *et al.*, 2008 ▸; Arunmanee *et al.*, 2014 ▸). According to these observations, the presence of free APol may be important for the long-term stability of MP–APol complexes.

Here, we utilized small-angle neutron scattering (SANS) as a powerful tool to study the structure of MP and APol in solution in order to understand the self-organization of MP–APol in the absence of free APol. SANS has been widely used to study the solution structure and interactions between MPs and detergent micelles in solution (Breyton *et al.*, 2013 ▸). It also allows us to understand the size, shape and interactions of biomolecules and polymers. Here, the contrast-variation technique enabled us to separately resolve both individual components within mixed complexes of MP–APol. Outer membrane protein F (OmpF), the major porin of the *E. coli* outer membrane, was used as the model MP. OmpF is a trimeric protein, with each monomer forming a 16-stranded β-barrel channel which allows the diffusion of small hydrophilic molecules across the bacterial envelope (Cowan *et al.*, 1992 ▸). Using SANS, we observed the association of OmpF–APol into long linear complexes and the APol redistribution which follows the removal of free APol.

## 2. Materials and methods   

### 2.1. Production of deuterated OmpF   

Deuterated OmpF was produced from *E. coli* BE3000 cells (Garavito & Rosenbusch, 1986 ▸). The cells were first adapted onto a hydrogenated, solid minimal medium plate; this was followed by growth on an 85% D_2_O minimal medium plate (Artero *et al.*, 2005 ▸). Once colonies had grown on the plate, selected larger colonies were grown in 50 ml 85% D_2_O minimal liquid medium. Once growth had been established overnight, these cells were inoculated at a 1:20 ratio into 2 × 50 ml fresh 85% D_2_O minimal liquid medium. This step was repeated three times in order to increase the initial growth rate. The cells were harvested by centrifugation at 8000*g* at room temperature and resuspended in 10 ml fresh 85% D_2_O minimal liquid medium. This cell culture was then inoculated into a 1.5 l bioreactor. Growth was monitored by measuring the OD_600_. When the OD_600_ reached 10.0, the cells were harvested by centrifugation at 8000*g* at 4°C for 10 min and the deuterated OmpF was purified as described previously by Lakey *et al.* (1985 ▸). OmpF was precipitated in cold ethanol and was resuspended in 20 m*M* sodium phosphate buffer pH 7.9, 100 m*M* NaCl, 0.5%(*v*/*v*) octyl-POE detergent. The contrast-match point was determined using a range of D_2_O concentrations, as described by Arunmanee *et al.* (2016 ▸)

### 2.2. Reconstitution of OmpF into amphipol   

The preparation of MP–APol complexes has previously been described by Zoonens *et al.* (2005 ▸). In brief, a stock of APol A8-35 at 20 mg ml^−1^ in water was stirred using a magnetic stirrer overnight at room temperature before use. The APol was added to detergent-solubilized OmpF at a 1:10(*w*:*w*) OmpF:APol ratio in 20 m*M* sodium phosphate buffer pH 7.9, 100 m*M* NaCl, 0.5%(*v*/*v*) octyl-POE. After incubation for 15 min at room temperature, detergents were removed by incubating the mixture with wet polystyrene Bio-Beads that had been pre-washed with methanol and deionized water at a 1:10(*w*:*w*) detergent:beads ratio at room temperature for 3 h. Removal of the polystyrene beads was achieved by centrifugation using an Eppendorf 5424 benchtop microcentrifuge at 20 000*g* for 5 min at room temperature.

### 2.3. Small-angle neutron scattering (SANS)   

#### 2.3.1. SANS sample preparation   

APol at 10 mg ml^−1^ in water was dialysed into 20 m*M* sodium phosphate pH 7.9, 100 m*M* NaCl in 100% D_2_O, whereas the OmpF–APol complexes were passed through a Superose 12 column pre-equilibrated with 20 m*M* sodium phosphate pH 7.9, 100 m*M* NaCl. The protein-containing fractions were concentrated using Vivaspin concentrators with a 10 kDa molecular-weight cutoff and then dialysed against the same buffers in 0%, 23.5%, 77% and 100% D_2_O. The final protein concentration in the sample was determined spectrophotometrically by measuring the absorbance at 280 nm.

#### 2.3.2. SANS data collection   

Data collection was performed on the SANS2D beamline at ISIS, Rutherford Appleton Laboratory, UK. This is a time-of-flight SANS instrument that uses a white-beam technique with neutrons of wavelengths from 1.75 to 16.5 Å. SANS data were recorded using two ∼1 × 1 m detectors; the further detector is 4 m from the sample, while the second detector is closer and offset to a higher angle, to give a combined *q* range from 0.0045 to 1.9 Å^−1^. Data fitting was only carried out to a *q* of ∼0.75 Å^−1^, where the signal had reached background. The samples (approximately 300 µl) were measured in 1 mm path-length quartz glass cuvettes at 20°C. Background data were also collected for the appropriate D_2_O/H_2_O mixtures. After allowing for the wavelength-dependent incident spectrum, sample transmission and detector efficiencies, the final reduced data were placed on an absolute scale by comparison with scattering from a partially deuterated polystyrene standard.

#### 2.3.3. Data analysis   

At the low sample concentrations with salt buffers used here, interparticle interactions should be minimal and the SANS intensity should be given by
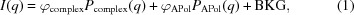
where there is a volume fraction φ of each component having form factor *P*(*q*) and we include a residual flat background (BKG) in the fits to compensate for any remaining discrep­ancy in the subtraction of incoherent and/or inelastic scattering from hydrogen. *q* = (4π/λ)sin(θ/2), where λ is the wavelength and θ is the scattering angle. The *P*(*q*) functions for shapes such as spheres, ellipsoids and cylinders are detailed in many standard texts on small-angle scattering. *P*(*q*) for ellipsoids and cylinders both require numerical integrations over the orientation angles of particles relative to *q*. For a uniform ellipsoid with axes *R*, *R* and *XR*, then
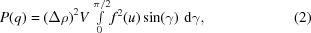
where *u* = *qR*(sin^2^γ + *X* ^2^cos^2^γ)^1/2^, *V* = (4π/3)*XR* ^3^ and *f*(*u*) = 3[sin(*u*) − *u*cos(*u*)]/*u* ^3^.

Δρ is the neutron scattering length density difference between particle and solvent. The scattering length density is the sum of tabulated scattering lengths *b_i_* divided by the volume *V* of the atoms involved. Owing to a phase shift, *b* is negative for hydrogen, so for example ρ for water varies between −0.56 × 10^−6^ Å^−2^ in H_2_O and +6.34 × 10^−6^ Å^−2^ in D_2_O. This means that Δρ can be made zero, *i.e.* ‘contrast matched’, for components such as lipids or surfactants at different water compositions.

For a cylinder of radius *R* and length *L*, the integral has
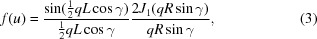
where *J*_1_(*x*) is a first-order Bessel function and now *V* = π*R* ^2^ *L*.

For core plus shell particles *f*(*u*) has terms for both core and shell and the volume normalization is slightly different. Given the correct scattering length densities and absolute scattering intensities, fitting programs such as *FISH* (Heenan, 2005 ▸) can provide volume-fraction estimates as well as determining the likely sizes and/or shapes of particles.

## 3. Results   

### 3.1. Self-assembly of APol in aqueous buffer determined by SANS   

**Figure 1:**
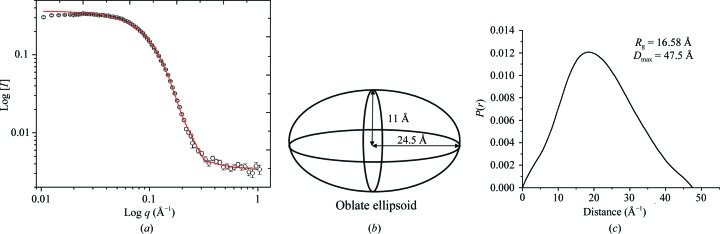
The scattering profile of amphipol A8-35 in D_2_O reveals the structure of amphipol A8-35 to be an oblate ellipsoid. (*a*) SANS data (symbols) and fitting (line) from *FISH*. (*b*) An oblate ellipsoid was the best-fitting simple uniform geometric shape model of free hAPol. (*c*) *P*(*r*) distribution function of free hAPol calculated by *GNOM*. APol was at 10 mg ml^−1^ in 20 m*M* sodium phosphate pH 7.9, 100 m*M* NaCl.

SANS is well adapted to determine the masses, shapes and dispersions of particles (Zaccaï & Jacrot, 1983 ▸). The solution structure of APol was investigated using SANS. APol was solubilized at 10 mg ml^−1^ in water and then dialysed into 100% D_2_O buffer (20 m*M* sodium phosphate pH 7.9, 100 m*M* NaCl). Initial data analysis by *GNOM* (Svergun, 1992 ▸) provided a *p*(*r*) distribution function that gave a radius of gyration (*R*_g_) of 16.6 Å and a maximum dimension (*D*_max_) of 47.5 Å (Fig. 1 ▸ *c*). The data were then analysed using the *FISH* modelling suite (Heenan, 2005 ▸). Here, an oblate ellipsoid (Fig. 1 ▸ *b*) with radii 11, 24.5 and 24.5 Å (which would give an *R*_g_ of 16.25 Å, in agreement with the *GNOM* analysis) provided the best fit to the experimental data (Fig. 1 ▸ *a*). Using the revised mean molecular mass for APol of 4 kDa (Giusti *et al.*, 2014 ▸), this result predicts that each particle of APol consists of ∼2.6 molecules. The *R*_g_ measured here is smaller than that measured previously (24 Å; Gohon *et al.*, 2006 ▸), but possible variations in size owing to the solution composition have been suggested (Giusti *et al.*, 2014 ▸).

### 3.2. OmpF–APol complexes studied by size-exclusion chromatography and small-angle scattering   

**Figure 2:**
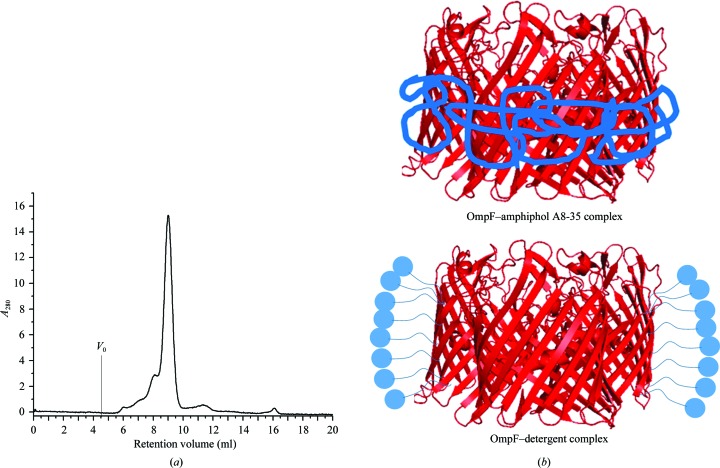
Size-exclusion chromatography shows monodisperse OmpF–amphipol A8-35 complexes. (*a*) Elution profile of monodisperse OmpF–amphipol A8-­35 complexes at a 1:10(*w*:*w*) ratio. SEC was carried out at a flow rate of 0.5 ml min^−1^ using a Superose 12 column equilibrated with 20 m*M* sodium phosphate pH 7.9, 100 m*M* NaCl. *V*_0_ represents the void volume of the column where aggregated proteins elute. Free APol is predicted to elute at 12 ml. (*b*) The proposed models of OmpF (red; PDB entry 2omp) in amphipol A8-35 and detergent micelles (blue). The OmpF structure is from the PDB with schematics of surrounding APol and detergent micelles.

The size of OmpF–APol complexes in detergent-free buffer was determined by size-exclusion chromatography (SEC) on a Superose 12 column (GE Healthcare). The elution profile in Fig. 2 ▸(*a*) indicated that the OmpF–APol complexes exiting the column were mainly monodisperse trimers (Fig. 2 ▸ *b*), with a very small amount of aggregate. Therefore, OmpF–APol complexes at a 1:10(*w*:*w*) OmpF:APol ratio (approximately a 1:100 molar ratio) are suitable to solubilize OmpF in the absence of conventional detergents. The elution profiles of APol show that free APol elutes at ∼12 ml; hence, the SEC results in the removal of free APol. Owing to this separation, the final OmpF:APol ratio in the protein-containing fraction is unknown. After removing free APol by SEC, the freshly eluted monodisperse OmpF–APol complexes assemble into 6 nm diameter filaments within an hour (Arunmanee *et al.*, 2014 ▸).

The structure of the OmpF–APol filaments was then studied by SANS using the contrast-variation technique, which requires knowledge of the accurate contrast-match point (CMP) of each component in the samples. The CMP is expressed as the %(*v*/*v*) of D_2_O where the scattering length density of the solvent is equal to that of the component and results in no observable scattering by that component. Deuterated OmpF (dOmpF) was produced as described and the CMP was experimentally determined to be 77%(*v*/*v*) D_2_O (Arunmanee *et al.*, 2016 ▸), whereas the CMP of APol (23.5%) has been reported by Gohon *et al.* (2004 ▸). The background contrast variation was achieved by preparing four OmpF–APol buffers containing different fractions of D_2_O so that the whole complex and individual components can be observed. The protein-containing fractions were collected and then dialysed (10 kDa cutoff) into an APol- and detergent-free buffer (20 m*M* sodium phosphate pH 7.9, 100 m*M* NaCl) containing 0%, 23.5%, 77% and 100% D_2_O. Both components of the complex scatter neutrons in 0% and 100% D_2_O buffer, whereas only dOmpF is visible in 23.5% D_2_O buffer, where APol is matched, and only APol is observed at the CMP of dOmpF in 77% D_2_O buffer. The final concentration of dOmpF in all samples was 2.02 mg ml^−1^; however, that of APol is unknown (the initial concentration of APol was 20 mg ml^−1^). The scattering data were recorded on the SANS2D beamline at ISIS, UK and were analysed using *FISH* (Heenan, 2005 ▸). The parameters used for the SANS data analysis are shown in Table 1 ▸.

**Figure 3:**
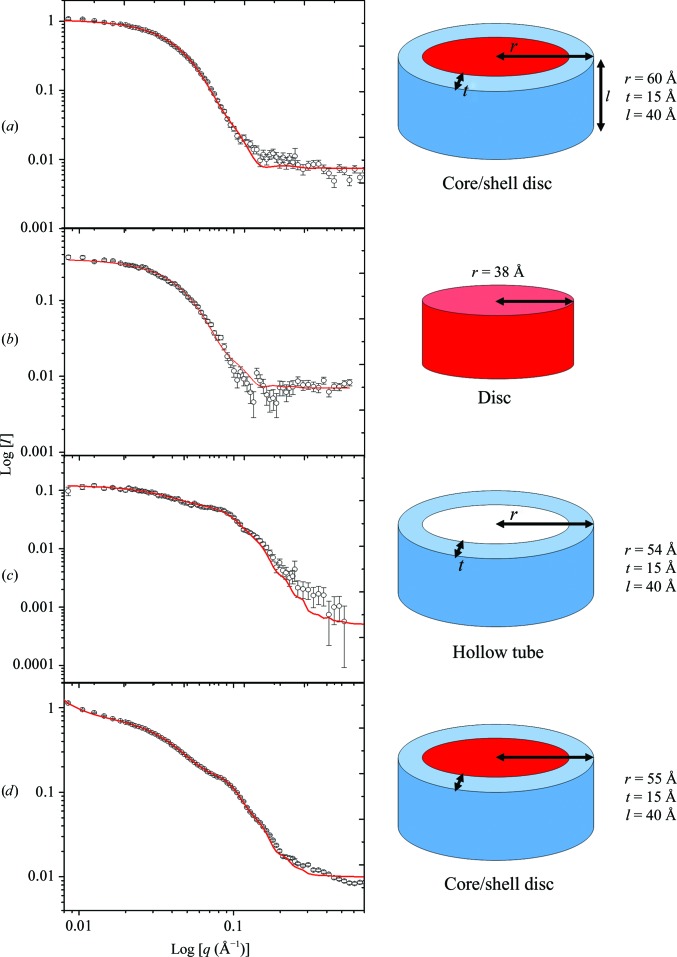
SANS data for OmpF–amphipol A8-35 complexes after removing free APol observed at different concentrations of D_2_O. The scattering data (symbols) and fitting (lines) using *FISH* with a core/shell tube for the complex, where OmpF is the core and APol is the shell, for (*a*) the sample in H_2_O and (*b*) the sample in 23.5% D_2_O, where APol is matched. (*c*) The sample in 77% D_2_O, where dOmpF is invisible to neutrons. (*d*) The sample in 100% D_2_O. In (*c*) and (*d*) a further small signal (dashed) is included for free APol ellipsoids. In (*d*) a *q* ^−3.5^ term for the up-turn at smallest *q* allows ‘filaments’.

**Figure 4:**
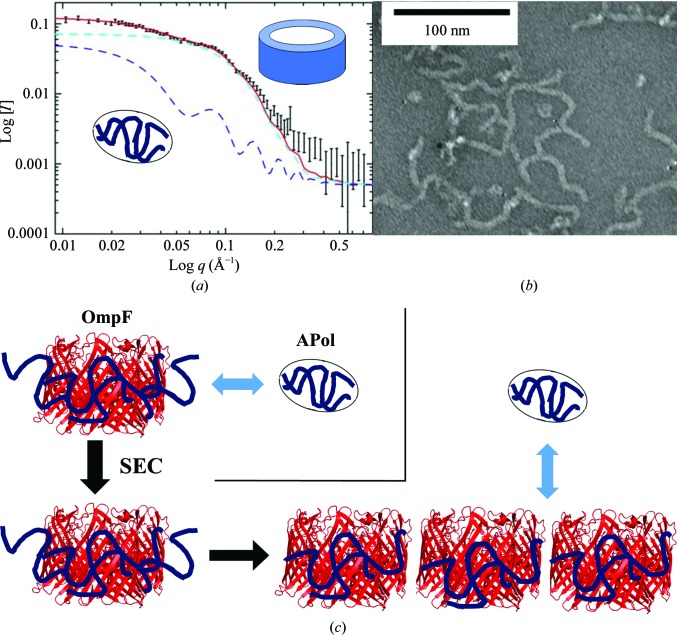
Model of the distribution of Apol in filaments. (*a*) The combination of fitted models required to fit the pure Apol scattering in 23.5% D_2_O. Core/shell tube data (dark blue dashed line) are combined with an oblate sphere model component (cyan dashed line) to provide a fit (red line) to the original data points. (*b*) Transmission electron microscopy image of OmpF–Apol filaments prepared as in Arunmanee *et al.* (2014 ▸), showing an absence of visible free Apol. (*c*) Schematic of the sequence of events leading to the formation of filaments and free APol. A variable inter-OmpF distance could explain the lack of long-range structure observed by SANS.

The scattering profiles and fitting of OmpF–APol at different concentrations of D_2_O are illustrated in Fig. 3 ▸. According to the crystal structure of OmpF (PDB entry 2omp; Cowan *et al.*, 1992 ▸; Fig. 2 ▸ *b*) its structure is disc-like, whereas the detergents or amphipols are bound to the hydrophobic region of OmpF located on the outside of the disc (Fig. 2 ▸ *b*). Hence, simple models representing OmpF and APol were chosen for the analysis. Fig. 3 ▸(*b*) shows the scattering curve and fitting of OmpF–APol complexes in 23.5% D_2_O, where only dOmpF is visible to neutrons. This data set fitted a disc model with a height of 40 Å and a radius of 49 Å, consistent with the known OmpF structure. This model also fits SANS data from dOmpF in contrast-matching SDS detergent (Clifton *et al.*, 2012 ▸; data not shown). The structure of APol in complex with OmpF was studied at 77% D_2_O, where dOmpF is invisible to neutrons. The scattering thus originates solely from the APol, and the red line fitted to this data in Fig. 3 ▸(*c*) results from a combination of hollow-tube and oblate ellipsoid models. The hollow tube with outer radius 54 Å, wall thickness 15 Å and height 40 Å (Fig. 3 ▸ *c*) represents APol in the complex, whereas the oblate ellipsoids represent free APol particles (Fig. 4 ▸ *a*). This is an indication that free APol is present in the filamentous samples but is invisible to EM (Fig. 4 ▸ *b*). As the free APol had previously been removed by SEC during sample preparation, the free APol observed in these samples must originate from Apol originally bound to the monodisperse complexes (Fig. 4 ▸ *c*). The SANS method does allow us to estimate that the amount of excess APol present is approximately 4 mg ml^−1^ in the 77% D_2_O sample. However, there is no sign of a filamentous structure of APol, which should appear as an upturn in the low-*q* range of the scattering data.

After the individual components of the complex had been resolved by SANS at the CMPs for APol and dOmpF, respectively, the components were combined using a core/shell tube model to represent the dOmpF–APol complexes which scatter at 0 and 100% D_2_O. dOmpF forms the core, whereas APol forms the surrounding shell. The scattering data of OmpF–APol in 0% D_2_O (Fig. 3 ▸ *a*) were fitted with the core/shell tube, but it was not necessary to include the free APol to obtain a good fit. A good fit is obtained from a model with shell width 15 Å, outer radius 60 Å and height 40 Å (Fig. 3 ▸ *a*; Table 1 ▸). The proximity to the CMP of Apol means that the scattering is dominated by OmpF.

However, oblate ellipsoids for free APol must be included in the fit for the complexes in 100% D_2_O. Fig. 3 ▸(*d*) shows the scattering data of complexes in 100% D_2_O. The fit is a combination of core/shell tube and oblate ellipsoids representing OmpF/APol complexes and free APol, respectively. In this case, the absolute SANS intensities suggest that roughly 5 mg ml^−1^ excess APol was found in the samples and that 1.4 mg ml^−1^ APol wrapped 2 mg ml^−1^ dOmpF. A core/shell tube (Fig. 3 ▸ *d*) fits this 100% D_2_O data with a shell width of 15 Å, an outer radius of 55 Å and a height of 40 Å. The 15 Å shell and 40 Å height are thus consistent across samples. The radius of free OmpF was determined to be 49 Å, so a total radius including the 15 Å shell would predict a radius of 64 Å. In the event, 0% D_2_O gives a result of 60 Å and 100% D_2_O gives a result of 55 Å. Intercalation of amphipol with the imperfect disc of OmpF may explain this lower figure.

The scattering curve of 100% D_2_O is the only curve that shows an upturn in the low-*q* region, included here as a *q* ^−3.5^ term. This may be indicative of a long-range structure or filament. All in all, the findings from the SANS study of OmpF–APol complexes indicated that the complexes consisted of OmpF wrapped by APol, but the filament structure was only seen in 100% D_2_O samples. Moreover, excess APol was found in the samples, even though it should have been removed by SEC during sample preparation or during dialysis. Thus, monodisperse OmpF–Apol complexes elute from the column and then undergo a re-equilibration with free amphipol (Fig. 4 ▸ *c*). The loss of amphipol from the individual complexes is compensated by the formation of filaments, in which protein–protein interactions may take the place of protein–amphipol interactions. The lack of filament signal in the SANS data for OmpF at 23.5% indicates that there is no clear long-range repetitive order of OmpF trimers in the fibres observed by transmission electron microscopy (TEM; Fig. 4 ▸).

It should be noted that the structural parameters chosen here, after some trial and error, from SANS are of ‘low resolution’ owing to the large number of potential parameters and the approximation of complex structures by simple geometric shapes with sharp interfaces and regions of uniform scattering. However, the four different contrasts studied present an entirely consistent view.

## 4. Discussion   

APols, a new class of detergents, have been used in a number of structural studies including NMR, SANS, EM *etc.* OmpF was reconstituted into APol with the aim of solubilizing and stabilizing OmpF in solution for molecular-interaction studies. Unexpectedly, instead of forming individual particles in solution, TEM data indicated that OmpF–APol assembled as filaments automatically after the removal of free APol by SEC (Arunmanee *et al.*, 2014 ▸). This self-association of MP–APol complexes when lacking free APol has also been reported by Zoonens *et al.* (2007 ▸) and Gohon *et al.* (2008 ▸). This suggested that free APol is essential for the stability of MP–APol complexes in solution. Here, SANS experiments on OmpF–APol complexes purified by SEC confirmed that some of the APol that was initially bound to monodisperse OmpF immediately after SEC dissociated from the complex to create a new pool of free APol. Once this fraction of the APol had been removed from the OmpF–Apol complexes, the remaining APol was not sufficient to keep OmpF monodisperse. Subsequently, the filaments start to assemble rapidly, presumably to minimize the hydrophobic surface exposed to the aqueous buffer. The model generated from the SANS data also suggests that APol wraps around OmpF in a similar way to conventional detergents, so that the removal of Apol increases the exposure of the hydrophobic belt. The SANS experiment on these complexes was unable detect the filamentous structure observed by EM; the complexes appeared as distinct core shell structures. An upturn in the low-*q* region is an indication of a filamentous structure, but this was only observed in the sample in 100% D_2_O. The lack of this feature could be owing to the fact that the scattering of free APols is stronger than that in the filaments or that it is difficult to see them in the *q*-range of the SANS2D instrument. The OmpF filaments are easily disrupted by adding lipopolysaccharide (LPS) to OmpF–APol complexes. LPS, a lipid found in the outer leaflet of Gram-negative bacteria, specifically binds to the hydrophobic belt of OmpF (Arunmanee *et al.*, 2016 ▸), suggesting again that the filaments are arranged as side-to-side strips of OmpF trimers. Interestingly, the addition of LPS leads to a sheet-like two-dimensional structure (Arunmanee *et al.*, 2014 ▸) which is reminiscent of the outer membrane of *E. coli* comprising OmpF and LPS. Thus, MP–Apol filaments may even provide a method of creating two-dimensional crystals for structural studies (Baboolal *et al.*, 2008 ▸; Arunmanee *et al.*, 2014 ▸), with the minimal remaining Apol acting as a crystallization chaperone.

## Figures and Tables

**Table 1:** Geometric parameters of amphipol A8-35 and OmpF–amphipol A8-35 complexes obtained by fitting SANS data Concentrations are estimated from SANS intensities. SLD, scattering length density.

		Fitting parameters							
Sample	Modelled shape	Concentration (mg ml^−1^)	SLD, core (10^−6^ Å^−2^)	SLD, shell (10^−6^ Å^−2^)	SLD, water (10^−6^ Å^−2^)	% water in particles	Radius *r* (Å)	Length *l* (Å)	Thickness *t* (Å)
APol A8-35	Oblate ellipsoid	APol, 10	1.06	N/A	6.35	46	11, 24.5, 24.5	N/A	N/A
OmpF–APol in H_2_O	Core/shell disc	Free APol, 0.01; APol shell, 1.43; OmpF, 2.32	4.81	1.06	−0.56	APol, 46; OmpF, 44	60	40	15
OmpF–APol in 23.5% D_2_O	Disc	APol-matched OmpF, 2.02	4.81	N/A	1.06	44	49	40	N/A
OmpF–APol in 77% D_2_O	Hollow tube	Free APol 4; APol shell, 0.93; OmpF, 1.27;	4.81	1.06	4.7607	APol, 46; OmpF, 44	54	40	15
OmpF–APol in 100% D_2_O	Core/shell disc	Free APol, 5.3; APol shell, 1.4; OmpF, 1.99	4.81	1.06	6.35	APol, 46; OmpF, 44	55	40	15
